# Ability of triglyceride‐glucose indices to predict metabolic dysfunction associated with steatotic liver disease in pediatric obesity

**DOI:** 10.1002/jpn3.70201

**Published:** 2025-08-22

**Authors:** Procolo Di Bonito, Anna Di Sessa, Maria Rosaria Licenziati, Domenico Corica, Małgorzata Gabriela Wasniewska, Anita Morandi, Claudio Maffeis, Maria Felicia Faienza, Enza Mozzillo, Valeria Calcaterra, Francesca Franco, Giulio Maltoni, Emanuele Miraglia del Giudice, Giuseppina Rosaria Umano, Giuliana Valerio

**Affiliations:** ^1^ Department of Internal Medicine “S. Maria delle Grazie” Hospital Pozzuoli Italy; ^2^ Department of the Woman, the Child and General and Specialized Surgery University of Campania “Luigi Vanvitelli” Napoli Italy; ^3^ Neuro‐Endocrine Diseases and Obesity Unit, Department of Neurosciences Santobono‐Pausilipon Children's Hospital Napoli Italy; ^4^ Department of Human Pathology of Adulthood and Childhood “G. Barresi”, Pediatric Unit University of Messina Messina Italy; ^5^ Department of Surgery, Dentistry, Pediatrics and Gynecology, Section of Pediatric Diabetes and Metabolism University and University Hospital of Verona Verona Italy; ^6^ Pediatric Unit, Department of Precision and Regenerative Medicine and Ionian Area University of Bari “A. Moro” Bari Italy; ^7^ Department of Translational Medical Science, Section of Pediatrics University of Napoli “Federico II” Napoli Italy; ^8^ Pediatric Department, “V. Buzzi” Children's Hospital, Milano and Department of Internal Medicine University of Pavia Pavia Italy; ^9^ Pediatric Department Azienda Sanitaria Universitaria del Friuli Centrale, Hospital of Udine Udine Italy; ^10^ Pediatric Unit, IRCCS Azienda Ospedaliero‐Universitaria di Bologna Bologna Italy; ^11^ Department of Medical, Movement and Wellbeing Sciences University of Napoli “Parthenope” Napoli Italy

**Keywords:** alanine aminotransferase, insulin resistance, liver ultrasonography

## Abstract

**Objectives:**

We aimed to evaluate the best cut‐off of alanine aminotransferase (ALT) to predict the metabolic dysfunction‐associated steatotic liver disease (MASLD) in youths with overweight/obesity (OW/OB) and analyze the performance of the triglyceride‐glucose index (TyG) or its association with anthropometric variables compared to ALT.

**Methods:**

This multicenter, cross‐sectional study analyzed data of 2813 youths (1463 boys and 1350 girls) aged 6–17 years recruited in 10 Italian centers for the management of pediatric OB. Exclusion criteria were: body mass index (BMI) *Z*‐score >5, diabetes, secondary obesity, triglycerides (TG) ≥ 400 mg/dL. MASLD was defined on the presence of hepatic steatosis at abdominal ultrasound, in combination with at least one cardiometabolic risk factor. The performance of ALT and TyG‐derived indices for MASLD diagnosis was assessed using the receiver operation curve and the area under the curve (AUC) with the 95% confidence intervals were obtained.

**Results:**

MASLD was observed in 1278 youths (45.1%). The global AUC of ALT was 0.661 (0.641–0.682), *p* < 0.0001. Using Youden's index the best cut‐off of ALT was ≥26 IU/L in boys and ≥22 IU/L in girls. The AUCs of TyG‐BMI, TyG‐WC, and TyG‐ALT were 0.678 (0.659–0.698), 0.659 (0.639–0.679), and 0.666 (0.645–0.686), respectively. Using Delong's test none TyG‐derived index showed a better performance than ALT alone.

**Conclusions:**

This study demonstrates that the values of ALT ≥ 26 IU/L in boys and ≥22 IU/L in girls can be used for the screening of MASLD in youths with OW/OB, while the combination of TyG‐derived indices is not superior to ALT alone.

## Abstract (highlights)

**What is Known:**

- Pediatric obesity is frequently associated with fatty liver that hampers the metabolic derangements in these subjects.
- Several noninvasive predictors of metabolic dysfunction‐associated steatotic liver disease (MASLD) have been proposed, however, no consensus has been reached.

**What is New:**

- Alanine aminotransferase (ALT) cut‐off levels for MASLD screening in normal‐weight youths can be applied to overweight (OW) individuals.
- The triglyceride‐glucose index (TyG) and TyG‐related parameters are not superior to ALT in detecting MASLD in youth with OW and obesity.

## 1 INTRODUCTION

Pediatric obesity (OB) has significant social and health consequences, both in industrialized and resource‐poor societies.^1^, ^2^ The World Health Organization (WHO) reported that the prevalence of overweight (OW) and OB in children and adolescents dramatically increased from 8% to 22% in the period 1990–2022.^3^ Obviously, the relevance of this phenomenon has a negative impact on several obesity‐related comorbidities, including nonalcoholic fatty liver disease (NAFLD).^4^, ^5^ The prevalence of NAFLD among youths with OB ranges between 35% and 45%.^6^ The strict association between these two conditions represents a dangerous cluster that may expose not only to the progression to liver fibrosis but also to the risk of cardiovascular events and diabetes later in life.^7^, ^8^ The accumulation of excessive intrahepatic fat has a multifactorial pathogenesis, such as genetic, environmental, and metabolic factors.^5^ Therefore, the new nomenclature “metabolic dysfunction–associated steatotic liver disease” (MASLD) was proposed instead of “NAFLD” to identify a condition characterized by the presence of hepatic steatosis and at least one cardiometabolic risk factor among abdominal adiposity, elevated blood pressure (BP), dyslipidemia, and/or dysglycemia.^9^, ^10^ The new definition seems to better reflect the association between metabolic risk factors and hepatic steatosis in view of the potential predictive role of the subsequent risk of metabolic and cardiovascular disease and provides a more comprehensive and effective framework, facilitating both early identification and supporting targeted treatment approaches.^9^, ^11^, ^12^

The North American Society of Pediatric Gastroenterology, Hepatology and Nutrition (NASPGHAN) recommended the evaluation of alanine‐amino transferase (ALT) as the first step for the screening of NAFLD in children, and sex‐specific cut‐offs were indicated (26 IU/L in boys and 22 IU/L in girls).^13^ Looking specifically at youths with OW/OB, higher cut‐offs that exceed twice the threshold have been found in children over 10 years of age, that is, 50 IU/L in boys and 44 in girls,^14^ but the question still remains controversial.^15^, ^16^ On the contrary, the European Society for Pediatric Gastroenterology, Hepatology and Nutrition (ESPGHAN) recommended using both ALT and abdominal ultrasound.^17^

Since insulin resistance (IR) is one of the most important metabolic factors associated with MASLD, the triglyceride‐glucose index (TyG) has been proposed as a biomarker of IR useful to identify subjects at risk of MASLD. Recent studies conducted mainly in adults from Asian countries (Chine and South Korea) showed that TyG or the products of its multiplication by anthropometric parameters, such as body mass index (BMI) (TyG‐BMI), BMI *Z*‐score (TyG‐zBMI), waist circumference (TyG‐WC), or waist to height ratio (TyG‐WHtR), exhibited a good performance assessed by area under curve (AUC) (between 0.760 and 0.860) in relation to the presence of NAFLD/MASLD.^18^ Similarly, Song et al. evaluated the usefulness of TyG‐indices in 3728 youths aged 10–19 years using high ALT as surrogate of NAFLD.^19^ In this study, the TyG‐BMI SDS, had better predictive power ([AUC: 0.683 [0.627–0.740]) than conventional TyG index (0.597 [0.538–0.656]), and insulin levels (0.635 [0.576–0.695]).^19^ As far as we know, no study compared the performance between the TyG index (or its variations based on anthropometric variables) and ALT in detecting NAFLD, while the product of TyG by ALT was tested by ultrasound fatty liver index with respect to the severity of NAFLD in children with OB.^20^

Based on these premises, the objectives of the present study are: to define the most appropriate cut‐off of ALT in relation to the presence of MASLD and whether the combination of TyG alone or combined with anthropometric variables can have a better performance than ALT alone to identify MASLD in a large multicentric cohort of youths with OW/OB.

## 2 METHODS

This multicenter, cross‐sectional study retrospectively included the data of 3175 young people observed from 2003 to 2016 across 10 Italian centers (three from northern, two from central, and five from southern Italy) dedicated to the management of OB enrolled in the “CARdiometabolic risk factors in OW and obese children in ITALY” (CARITALY) study on the behalf of the Obesity study group of the Italian Society for Pediatric Endocrinology and Diabetology. Three hundred‐sixty‐two individuals were excluded from the subsequent analyses because they did not meet the inclusion criteria. Therefore, the data of 2813 youths (1463 boys and 1350 girls) were analyzed (103 with OW and 2710 with obesity).

The inclusion criteria were (a) age between 6 and 17 years, (b) BMI > + 1 *Z*‐score according to the WHO growth standards, and (c) availability of complete anthropometric, clinical, and biochemical data. Exclusion criteria were: BMI *Z*‐score >5, type 1 or type 2 diabetes, secondary forms of obesity or of hepatic steatosis, serum low‐density lipoprotein‐cholesterol (LDL‐C) levels ≥190 mg/dL, triglycerides (TG) ≥ 400 mg/dL, presence of other chronic diseases, or chronic use of medication potentially leading to metabolic disturbance (e.g., steroids).

### 2.1 Ethics statement

The study was approved by the Research Ethical Committee of University of Campania “Luigi Vanvitelli” (protocol code 834/2016) and was conducted in accordance with the ethical guidelines of the 1975 Declaration of Helsinki and its later amendments or comparable ethical standards. A written informed consent was obtained before any procedure.

### 2.2 Clinical evaluations

At each center, trained physicians conducted physical examinations, and anthropometric measurements were obtained following standardized procedures according to Italian recommendations.^21^ Pubertal maturation was assessed according to Tanner stages. BMI was calculated as weight (kg) divided by height (cm) squared. BMI *Z*‐scores were calculated using the WHO AnthroPlus software.^22^ WC was measured at the midpoint between the last rib and the iliac crest during minimal respiration while the participant was in an upright position. Waist‐to‐height ratio (WHtR) was also calculated as previously described.^23^

BP was measured three times, with a 2‐min interval between each measurement, in a seated position after a 5‐min rest, using an appropriately sized aneroid sphygmomanometer. The mean of the last two measurements was calculated and used for analysis.^23^

### 2.3 Laboratory evaluations

After an overnight fast, blood samples for glucose, insulin, transaminase, total cholesterol, TG, high density lipoprotein‐cholesterol (HDL‐C), were collected. Low‐density lipoprotein‐cholesterol (LDL‐C) level (mg/dL) was calculated using the Friedewald equation. Homeostatic model assessment of IR (HOMA‐IR) was calculated as: fasting insulin (mLU/L) × fasting glucose (mg/dL)/405. The TyG index was calculated as it follows: Ln fasting TG (mg/dL) × fasting glucose (mg/dL)/2. The other TyG‐derived indices were calculated accordingly: TyG × BMI (TyG‐BMI); TyG × zBMI (TyG‐zBMI); TyG × WC (TyG‐WC); TyG × WHtr (TyG‐WHtR); TyG × ALT (TyG‐ALT).

Analysis of all biochemical parameters was performed in the centralized laboratory of each center, as previously described.^23^ Glucose, TG, HDL‐C, and ALT were analyzed using colorimetric method, while plasma insulin was analyzed by immune‐enzymatic method. All participating laboratories were part of the Italian National Health System and were certified according to ISO 9000 international standards (www.iso9000.it/) with semiannual quality control and inter‐laboratory comparisons.

### 2.4 Liver ultrasound evaluation

Liver ultrasonography was performed by trained radiologists at each center to detect the presence of hepatic steatosis (as present or absent). Hepatic steatosis was defined by the bright liver sign compared to the right kidney, as previously described.^23^

### 2.5 Definition of MASLD

MASLD diagnosis was made according to a multisociety Delphi consensus statement^10^ However, since we studied a population of youths with OW/OB, diagnosis of MASLD was based on the presence of ultrasound‐detected hepatic steatosis, in combination with at least one of the following cardiometabolic risk factors: (1) WC ≥95th percentile; (2) high BP: age <13 years: BP ≥95th percentile for age and sex and height, or age ≥13 years BP ≥ 130/85 mmHg; (3) high plasma TG (<10 years: ≥100 mg/dL; ≥10 years: ≥150 mg/dL), (4) low HDL‐C (≤40 mg/dL), (5) fasting glucose ≥100 mg/dL or plasma glucose ≥200 mg/dL or 2 h postload glucose ≥140 mg/dL or HbA1c ≥ 5.7%.^10^, ^24^

### 2.6 Statistical analysis

The normality of the variables was assessed using the Kolmogorov–Smirnov test. Continuous variables with a normal distribution are presented as means ± standard deviation or standard error (SE), whereas those with a skewed distribution were log‐transformed before analysis and presented as medians with interquartile ranges (IQR). Between‐group comparisons for continuous variables were performed using Student's *t*‐test. Qualitative variables are expressed as number and percentages (%), with comparisons made using the *χ*² test.

The diagnostic performance of ALT versus MASLD was performed using receiver operating characteristic (ROC) curve analysis. AUC values, along with their 95% confidence intervals (95% CI), were calculated separately in the two sexes and the Youden's index was used to determine the optimal cut‐off point. The AUC was interpreted as it follows: 0.5 < AUC ≤ 0.7 low accuracy; 0.7 < AUC ≤ 0.9 moderate accuracy; 0.9 < AUC ≤ 1.0 high accuracy. The DeLong's test was applied to assess the statistical differences between AUCs.

The performance of the cut‐off value from high ALT levels and elevated values of TyG‐derived indices was evaluated by calculating sensitivity, specificity, positive predictive value (PPV), and negative predictive value (NPV), which were obtained using 2 × 2 contingency tables. A sensitivity or specificity cutoff of 0.7 was considered a minimal acceptable standard for a screening test.

A *p*‐value < 0.05 was considered for statistical significance. Statistical analyses were performed using IBM SPSS Statistics software, Version 28.0 (IBM Corporation).

## 3 RESULTS

The characteristics of youths without or with MASLD are presented in Table 1. Youths with MASLD were 1278 (45.1%) (721 boys and 629 girls) and showed higher age, higher prevalence of male sex, and higher values of all anthropometric and biochemical variables analyzed except for fasting glucose and cholesterol compared to those without MASLD.

**Table 1:** Characteristics of youths without or with MASLD in the overall sample.

	No MASLD	MASLD	
*n* = 2813	1535 (54.9%)	1278 (45.1%)	*p* value
OW/OB	86/1449	17/1261	<0.0001
Male sex, *n* (%)	742 (48.3)	721 (56.4)	<0.0001
Prepubertal, *n* (%)	408 (26.6)	235 (18.4)	<0.0001
Age, years	10.8 ± 2.6	11.3 ± 2.4	<0.0001
BMI (kg/m^2^)	28.7 ± 4.6	31.7 ± 5.2	<0.0001
BMI‐*Z* score	3.2 ± 0.9	3.5 ± 0.9	<0.0001
WC (cm)	90.5 ± 11.7	97.6 ± 13.4	<0.0001
WHtR (cm)	0.62 ± 0.07	0.65 ± 0.07	<0.0001
G_0_ (mg/dL)	86.4 ± 9.0	85.7 ± 9.4	0.032
HOMA‐IR	3.1 (2.1‐4.4)	3.6 (2.6‐5.2)	<0.0001
Cholesterol (mg/dL)	155.0 ± 28.5	155.9 ± 30.8	0.408
HDL‐C (mg/dL)	48.8 ± 11.4	46.2 ± 10.4	<0.0001
TG (mg/dL)	76.0 (58.0–105.0)	86.0 (63.0–117.0)	<0.0001
ALT (IU/L)	20.0 (16.0–26.0)	26.0 (19.0–40.0)	<0.0001
TyG	8.1 ± 0.4	8.2 ± 0.4	<0.0001
TyG‐BMI	232.9 ± 41.5	260.3 ± 46.4	<0.0001
TyG‐zBMI	25.6 ± 7.5	29.1 ± 7.4	<0.0001
TyG‐WC	735.0 ± 107.9	802.2 ± 123.7	<0.0001
TyG‐WHtR	5.0 ± 0.6	5.3 ± 0.7	<0.0001
TyG‐ALT	160.0 (126.3–212.5)	214.7 (151.9–324.9)	<0.0001

*Note*: Data are expressed as mean ± SD, number (%), median (interquartile range).

Abbreviations: ALT, alanine‐aminotransferase; BMI, body mass index; G_0,_ fasting glucose; HDL‐C, high‐density lipoprotein‐cholesterol; HOMA‐IR, Homeostatic Model Assessment for Insulin Resistance; MASLD, metabolic dysfunction‐associated steatotic liver disease; OB, obesity; OW, overweight; TG, triglycerides, Tyg, triglycerides‐glucose index; WC, waist circumference; WHtR, waist to height ratio.

**Figure 1:**
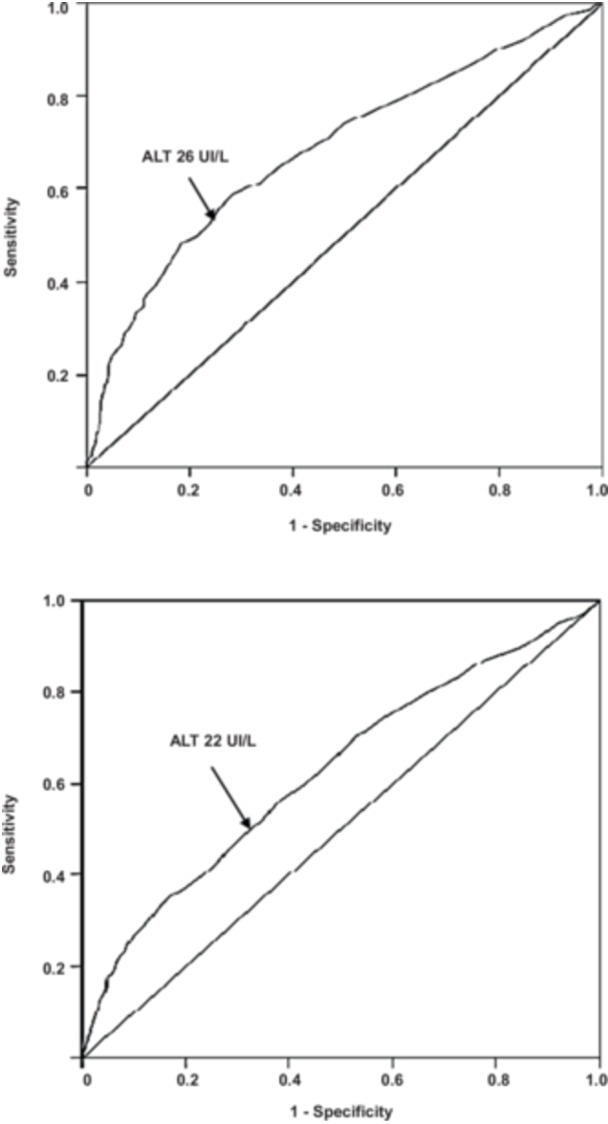
Area under curve of ALT for metabolic dysfunction‐associated steatotic liver disease in boys and girls. The upper panel displays data for boys and the lower panel data for girls. ALT, alanine aminotransferase.

The accuracy of ALT to classify the presence of MASLD was low (AUC 0.661, 95% CI 0.641–0.682, SE 0.010, *p* < 0.0001) in the overall sample; similar values were found in boys (AUC 0.683, 95% CI 0.657–0.710, SE 0.013, *p* < 0.0001) and girls (AUC 0.629, 95% CI 0.599–0.659, SE 0.015, *p* < 0.0001). Using Youden's index the best cut‐off of ALT was 26 IU/L in boys and 22 IU/L in girls (Figure 1). Children and adolescents with ALT values above this cut‐off were 1153 (41%) in the overall sample.

Subsequently, we assessed the accuracy of TyG alone or TyG combined with several anthropometric and biochemical variables to classify the presence of MASLD in the whole sample and by sex subgroups (Table 2). Generally, the AUCs were lower in girls than boys. The best AUC was obtained using TyG‐BMI, which reached a moderate accuracy of classification in boys (0.702), compared to the whole sample (0.678) and to girls (0.653). However, the AUC of TyG‐BMI was not significantly higher than that obtained by using ALT in boys (*p* = 0.380) and in girls (*p* = 0.216). Sensitivity, specificity, PPV, and NPV of high levels of ALT or TyG‐derived indices vs MASLD are compared in Table 3. All indices performed poorly: sensitivity spanned from 0.439 for TyG‐zBMI to 0.638 for TyG‐WHtR; specificity spanned from 0.569 for TyG‐WHtR to 0.722 for TyG‐zBMI.

**Table 2:** AUC of alanine‐aminotransferase and TG‐glucose index derived indices respect to metabolic dysfunction‐associated steatotic liver disease in the overall sample and separately by sex.

Variables	Cut‐off	AUC (95% Cl)	SE	*p* value
Overall sample (*n* = 2813)				
TyG‐BMI	≥239 in boys and ≥252 in girls	0.678 (0.659–0.698)	0.010	<0.0001
TyG‐ALT	≥213 in boys and 164 in girls	0.666 (0.645–0.686)	0.010	<0.0001
ALT	≥26 in boys and ≥22 in girls	0.661 (0.641–0.682)	0.010	<0.0001
TyG‐WC	≥782 in boys and 716 in girls	0.659 (0.639–0.679)	0.010	<0.0001
TyG‐zBMI	≥24 in boys and girls	0.641 (0.621–0.661)	0.010	<0.0001
TyG‐WHtR	≥5 in boys and ≥5.2 in girls	0.635 (0.614–0.655)	0.010	<0.0001
TyG	≥8.09	0.562 (0.541–0.583)	0.011	<0.0001
Boys (*n* = 1463)				
TyG‐BMI	≥239	0.702 (0.676–0.729)	0.013	<0.0001
TyG‐ALT	≥213	0.689 (0.662–0.716)	0.014	<0.0001
ALT	≥26	0.685 (0.658–0.712)	0.014	<0.0001
TyG‐WC	≥782	0.642 (0.639–0.697)	0.014	<0.0001
TyG‐zBMI	≥24	0.621 (0.592–0.649)	0.015	<0.0001
TyG‐WHtR	≥5	0.629 (0.601–0.658)	0.014	<0.0001
TyG	≥8.09	0.572 (0.543–0.601)	0.015	<0.0001
Girls (*n* = 1350)				
TyG‐BMI	≥252	0.653 (0.624–0.682)	0.015	<0.0001
TyG‐ALT	≥164	0.631 (0.601–0.661)	0.015	<0.0001
ALT	≥22	0.626 (0.596–0.657)	0.016	<0.0001
TyG‐WC	≥716	0.639 (0.610–0.669)	0.015	<0.0001
TyG‐zBMI	≥24	0.651 (0.622–0.681)	0.015	<0.0001
TyG‐WHtR	≥5.2	0.638 (0.608–0.668)	0.015	<0.0001
TyG	≥8.09	0.555 (0.524–0.586)	0.016	0.001

Abbreviations: ALT, alanine‐aminotransferase; AUC, area under curve; BMI, body mass index; CI, confidence interval; G_0,_ fasting glucose; HDL‐C, high‐density lipoprotein‐cholesterol; SE, standard error; TG, triglycerides, TyG, triglycerides‐glucose index; WC, waist circumference; WHtR, waist to height ratio.

**Table 3:** Performance of high levels of alanine‐aminotransferase and TG‐glucose index derived indices respect to metabolic dysfunction‐associated steatotic liver disease in the overall sample.

Variables	Sensitivity	Specificity	PPV	NPV
ALT ≥ 26 in boys and ≥22 in girls	0.538 (0.520–0.557)	0.697 (0.680–0.714)	0.597 (0.578–0.615)	0.645 (0.627–0.662)
TyG‐BMI ≥ 239 in boys and ≥252 in girls	0.593 (0.575–0.611)	0.675 (0.657–0.692)	0.603 (0.585–0.621)	0.666 (0.648–0.683)
TyG‐WC ≥ 782 in boys and ≥716 in girls	0.628 (0.609–0.645)	0.591 (0.572–0.609)	0.561 (0.542–0.579)	0.656 (0.638–0.673)
TyG‐zBMI ≥ 24 in either sex	0.439 (0.421–0.458)	0.722 (0.705–0.739)	0.568 (0.550–0.587)	0.607 (0.589–0.625)
TyG‐WHtR ≥ 5.0 in boys and ≥5.2 in girls	0.638 (0.620–0.656)	0.569 (0.551–0.588)	0.552 (0.534–0.571)	0.654 (0.636–0.672)
TyG‐ALT ≥ 213 in boys and ≥164 in girls	0.599 (0.581–0.618)	0.644 (0.626–0.662)	0.584 (0.565–0.602)	0.659 (0.641–0.676)

Abbreviations: ALT, alanine‐aminotransferase; BMI, body mass index; G_0,_ fasting glucose; HDL‐C, high‐density lipoprotein‐cholesterol; NPV, negative predictive value; PPV, positive predictive value; TG, triglycerides, TyG, triglycerides‐glucose index; WC, waist circumference; WHtR, waist to height ratio.

## 4 DISCUSSION

The present study demonstrates that the values of ALT ≥ 26 IU/L in boys and ≥22 IU/L in girls, as proposed by NASPGHAN for the screening of NAFLD in normal‐weight children and adolescents, are superimposable to that observed in our population of youths with OW/OB with respect to MASLD.

Currently, there is no agreement on the screening for MASLD in children at risk. In particular, the use of liver ultrasound is questioned.^15^ Given the limitation of transaminases in predicting steatotic liver disease in children with OW/OB, ALT does not appear to provide a better sensitivity and specificity for MASLD screening even when compared to TyG and its associations with anthropometric variables.

Other studies have reported the ability of TyG and its derived parameters to predict NAFLD and metabolic comorbidities in different cohorts.^25^, ^26^, ^27^, ^28^ A recent meta‐analysis reported an increase of 2.84‐fold NAFLD risk for each unit increase in TyG in adults, suggesting the predictive role of TyG for NAFLD. However, no specific TyG cut‐off was proposed and no comparison with other predictors was performed.^25^

In light of the recent shift in nomenclature from NAFLD to MASLD and its close pathophysiological association with dysmetabolism,^29^ research has also explored the potential role of the TyG index and its derived parameters in predicting MASLD, particularly in adults,^18^, ^26^, ^30^, ^31^ while similar evidence is still limited in pediatric age.^18^, ^32^ Despite the current lack of specific diagnostic cut‐offs, some studies supported the effectiveness of TyG as a noninvasive marker for predicting MASLD with good accuracy.^18^

Since TyG is calculated using fasting plasma glucose and TG, it is easily obtainable in clinical settings, facilitating its rapid application in clinical practice. In line with findings in adults, the TyG index has also emerged as a reliable, simple, noninvasive, and cost‐effective surrogate marker of IR, demonstrating a strong correlation with both cardiovascular and metabolic impairments in childhood.^33^, ^34^, ^35^, ^36^ Given its close pathophysiological link with IR—a key driver of multiple cardiometabolic disorders (e.g., MASLD, T2D, metabolic syndrome, cardiovascular disease, etc.)—the TyG index may be useful for identifying broader cardiometabolic risk and improving risk stratification in children with obesity.^36^ However, given the limited number of studies, further research on TyG and TyG‐derived parameters is necessary to evaluate their full potential. In addition, scientific evidence suggests that variables such as gender, disease type, ethnicity, sample size, degree of OW and OB, and age might affect the TyG performance and cut‐off, leading to data heterogeneity.^18^, ^26^

In our study, we found no significant improvement in MASLD prediction by using a combination of ALT and TyG, or its associations with anthropometric parameters compared to ALT alone. Even though TyG‐BMI had the highest AUC, it did not have greater clinical significance, as no index had an AUC > 0.70.

This finding contrasts with what has been described by other researchers.^25^, ^26^, ^27^, ^28^ However, this difference might be attributed to the peculiarity of our cohort which includes only children and adolescents with OW/OB. Considering that TyG can be considered as a surrogate index of IR, it might not contribute to MASLD prediction among patients with OW/OB who are also insulin resistant, as for our cohort. Moreover, in the study by Arslanian et al., TyG differed among pediatric patients with OB and different glucose tolerance status without including the evaluation of liver steatosis.^37^ Therefore, the utility of this index should be more deeply investigated in cohorts with known different degrees of IR and obesity‐related comorbidities.

Conversely, we found a substantial role of ALT levels in the discrimination of MASLD. The cut‐off of ALT, previously recommended by NASPGHAN for the screening of NAFLD and corresponding to 95^th^ percentile of ALT in healthy weight, metabolically normal, liver disease‐free individuals, was reported in the Screening ALT For Elevation in Today's Youth (SAFETY) study.^38^ Interestingly, our results underlined that this cut‐off may be applied even in youths with OW/OB and MASLD. In the study of Johansen et al. the ALT predictive cut‐off for the presence of liver steatosis was 34.5 IU/L in boys and 24.5 IU/L in girls with an AUC of 0.79 and 0.72, respectively.^39^ A different cut‐off was proposed in the study by Yu and coworkers, on 408 children and adolescents with OW and OB who underwent an abdominal magnetic resonance imaging (MRI).^4^ The authors found an ALT cut‐off of 42 IU/L in boys and 30 IU/L in girls for the presence of NAFLD.^4^ In our cohort, we found that an ALT cut‐off of 26 IU/L in boys and 22 IU/L in girls was predictive of MASLD. The differences in the optimal cut‐offs might be related to the heterogeneity of the cohorts included in the studies (normal weight and/or OW/OB), the hepatic steatosis assessment method (MRI vs*.* ultrasound), and the clinical outcome (NAFLD vs*.* MASLD). However, we should recognize that, albeit significant, this ALT cutoff displays a limited accuracy in the detection of MASLD with a sensitivity of 54% and a negative predictive value of 70%. Therefore, MASLD might be missed in at‐risk children by relying on ALT values alone, in agreement with a recent comparative study.^15^

As far as we know, our study is among the first to address the predictivity of ALT versus TyG‐derived indices for pediatric MASLD in a cohort of youths with OW and OB. A major strength of this study was the large sample size and comprehensive phenotyping of children, that allowed us to perform subanalyses by sex. Moreover, the participants were drawn from different tertiary centers reducing the selection bias that could arise from recruiting solely one referral center.

However, some limitations should be acknowledged, including the cross‐sectional design and the definition of MASLD by ultrasound, which does not provide an accurate quantification of fat accumulation compared to MRI. Since clinical and biochemical data were collected over a 15‐year period, possible heterogeneity in methodologies, tools, diagnostic interpretations, staff training cannot be excluded.

## 5 CONCLUSION

The validity of previously sex‐specific cut‐offs of ALT (26 IU/L in boys and 22 IU/L in girls) in children with NAFLD is confirmed also in youths with OW/OB and MASLD. Furthermore, our data do not support the superiority of biochemical predictors as TyG, or TyG‐derived factors respect to ALT for the screening of MASLD in children and adolescents with OW/OB.

Lacking predictive biomarkers, our results suggest the need for liver ultrasound to detect the presence of MASLD in youths with OW/OB. Further studies based on more accurate imaging modalities might contribute to assess whether the evaluation of ALT associated with other biomarkers may help to discriminate more severe forms of steatotic liver disease.

## CONFLICT OF INTEREST STATEMENT

The authors declare no conflict of interest.
